# Impact of ripening stages and drying techniques on the physicochemical and sensory attributes of apple mango chips

**DOI:** 10.1111/1750-3841.17585

**Published:** 2025-01-13

**Authors:** Messenbet Geremew Kassa, Desye Alemu Teferi

**Affiliations:** ^1^ College of Agriculture, Food, Climate Science Injibara University Injibara Ethiopia

**Keywords:** chips, drying methods, physicochemical properties, ripening stages, sensory quality

## Abstract

Mangoes (*Mangifera indica* L.) are valued for their rich nutrients, including vitamins A, B, C, carotenoids, and phenolic compounds. However, high moisture content and seasonal availability contribute to post‐harvest losses of up to 50%. To reduce these losses and extend shelf life, drying methods like solar, convective, and freeze‐drying are used, each impacting dried mango quality differently. This study examines how ripening stages and drying methods affect the physicochemical and sensory qualities of apple mango chips, analyzing attributes such as moisture (7.81%–11.50%), protein (2.49%–2.89%), fat (0.78%–1.11%), pH, total soluble solids, and color parameters (L* and a* values). Results show that solar drying, especially for fully ripe mangoes, enhances color and sensory qualities, with fully ripe, solar‐dried mango chips receiving the highest ratings for color, taste, flavor, and aroma. This emphasizes the importance of optimizing ripeness and drying techniques to improve dried mango quality. Although solar drying is cost‐effective and preserves sensory qualities, limitations include nutrient loss and limited access to advanced drying technologies like freeze‐drying, especially in developing regions. The study's focus on apple mangoes and a small, non‐trained sensory panel may limit generalizability, suggesting that future research could explore additional drying methods across more mango varieties. Larger sensory panels with trained evaluators may provide broader insights. This study offers valuable strategies for enhancing dried mango production and marketability. By optimizing ripening stages and drying techniques, industry professionals and researchers can improve product quality, meet consumer preferences, and support sustainability, benefiting local farmers and global markets.

## 1 INTRODUCTION

A healthy diet must include fruits and vegetables due to their essential vitamins, minerals, antioxidants, carbohydrates, and fiber, which are crucial for optimal nutrition and health (Dhandevi & Jeewon, 2015; Habauzit et al., 2014). Mangoes (*Mangifera indica* L.), known for their rich nutritional profile and diverse phytochemical content, rank third in global demand for tropical fruits, with over 55 million tonnes produced in 2019 (FAO, 2020; Manhongo et al., 2021). Often referred to as the “king of fruits,” mangoes are prized for their attractive colors, tender flesh, and robust flavor, which results from naturally occurring sugars like glucose, fructose, and sucrose, as well as flavor compounds such as car‐3‐ene and cis‐ocimene (Duyen et al., 2023; Lebaka et al., 2021; Liu et al., 2020; Scutigliani & Kikkert, 2017).

In developing countries, the demand for high‐value agro‐food products, including fruits and vegetables, is increasing in developing countries, often distributed through multilevel marketing schemes (Joosten et al., 2015; van Berkum, 2021). In Ethiopia, mango production is challenged by high post‐harvest losses (31.8%) and inconsistent measurement practices, which affect farmers' revenues and market performance production (Mossie et al., 2023). This is exacerbated by the disproportionate share of consumer prices received by district‐level collectors compared to farmers (40.18% vs. 15.08%), discouraging high‐quality production (Mossie et al., 2023). The seasonal nature of mangoes results in significant post‐harvest losses (30%–50%), necessitating effective preservation methods to reduce spoilage (Ekka & Mjawa, 2020). Various drying methods, including solar, convective, freeze, vacuum, osmotic, microwave, and hybrid techniques, are employed to extend the shelf life of fruits by reducing moisture content and slowing degradation (Aravindh & Sreekumar, 2015; Bisht et al., 2024; Dereje & Abera, 2020a; Olaniran et al., 2020). Drying is essential for preserving the nutritional and sensory qualities of fruits, as it inhibits enzyme activity and microorganism growth, thereby enhancing storage and transportation efficiency (Calín‐Sánchez et al., 2020; Mahiuddin et al., 2018). Optimizing drying techniques is crucial to minimizing nutrient loss and maintaining sensory qualities, such as color, aroma, taste, and texture, which significantly influence consumer acceptance (Abano, 2010; Pham et al., 2020; Sulistyawati et al., 2020; Sun et al., 2019; Yi et al., 2017).

Apple mangoes, specifically, have a high moisture content (above 82%), leading to a short shelf life and necessitating effective preservation methods (Bekele et al., 2020). Modern drying techniques, such as infrared (IR), vacuum, and freeze‐drying, offer advancements in fruit processing by improving handling, packaging, and transport while extending shelf life and maintaining product quality (Moraga et al., 2012; Salehi et al., 2017; Vanamala et al., 2005). For example, increasing hot air temperature and IR lamp power significantly reduces drying time for persimmons, demonstrating the efficacy of advanced drying methods (Salehi et al., 2017). Additionally, combining sonication with edible coatings enhances the quality of dried fruits. Sonication improves water diffusivity, reduces drying time, and increases total phenolic content and antioxidant activity in infrared‐dried fruits (Salehi, Inanloodoghouz, Amiri, et al., 2023; Salehi & Inanloodoghouz, 2023). Infrared drying is gaining popularity for its high efficiency and energy savings, while ultrasound‐assisted osmotic dehydration improves mass transfer kinetics, enhancing soluble solids gain and water loss (Ma et al., 2021; Meena et al., 2022). Microwave drying has been shown to improve the rehydration rate and maintain high levels of total sugars and ascorbic acid in dried fruits (Nayi et al., 2023; Salehi, Inanloodoghouz, Ghazvineh, et al., 2023). Despite the benefits of drying, some nutrients, such as vitamin C, can deteriorate during the process (Chang et al., 2016; Mahiuddin et al., 2018). Selecting appropriate drying methods is crucial for minimizing quality loss and enhancing the sensory attributes of dried apple mango chips. This study aims to evaluate the impact of ripening stages and drying techniques on the physicochemical and sensory properties of these chips, addressing a notable gap in research. While previous studies have explored the effects of ripening and various drying techniques on fruit quality, they have largely overlooked apple mangoes and the combined influence on sensory attributes like taste, flavor, and aroma. To address these gaps, this study investigates how different ripening stages and drying methods affect apple mango chips. Given the high cost of advanced drying technologies, practical and cost‐effective methods like solar drying and oven‐drying were chosen, with recommended drying temperatures employed to achieve optimal results within local resource constraints. The study on apple mango chips has significant national and international implications. Locally, it improves fruit processing, boosts market value, and aids food manufacturers and farmers in increasing profits. Globally, it enhances the competitiveness of apple mango producers, contributes to sustainable practices, and aligns with trends toward healthier snacks. The research also sets a benchmark for future studies in fruit processing technology.

## 2 MATERIALS AND METHODS

### 2.1 Sample collection

High‐quality raw apple mangoes were harvested from the Adeiet Research Center, Weramit Sub Research Center, located in Bahirdar, Ethiopia. The fruits were free from any damage, insect infestation, disease, or deterioration. They were then transported to the Food Science and Post‐harvest Technology Department at Injibara University, Ethiopia.

### 2.2 Sample preparation

The sample preparation for this study was based on Appiah et al. (2011), 144 mature apple mango fruits were selected from the same apple varieties but different trees, ensuring uniform size, undamaged condition, and no visible infection symptoms. The fruits were cleaned and divided into three groups of 48 based on ripening stages: unripe, intermediate, and fully ripe. Each group was further split into two subgroups of 24 fruits each, designated for solar drying and oven‐drying, respectively. These subgroups were divided into three replicates of eight fruits each. The fruits ripened at room temperature (30–33°C). Ripening stages were classified as follows: unripe (firm with no thumb depression), intermediate (slight depression), and fully ripe (strong aroma with noticeable indentation). This procedure was repeated for all treatments to ensure consistent ripening stages.

### 2.3 Chips production

Fresh apple mangoes were given a quick rinse to remove any surface dirt. Wrapped in plastic, they took a hot bath (90°C) for 2 min to deactivate enzymes. A plunge into an ice bath (7°C) stopped them from cooking further. After peeling and splitting the apple mangoes in half, the pits were removed. The flesh was sliced into thin strips measuring approximately 2 cm × 4 cm × 0.5 cm. One batch of these slices was sun‐dried for 5 days at temperatures exceeding 25°C. The other batch was dried in a warm sauna set at 60°C for 12 h, resulting in a similar level of dehydration. Finally, the dried apple mango chips were packed in plastic bags and placed in a special container (desiccator) to keep them fresh (Appiah et al., 2011).

### 2.4 Juice preparation from apple mango chips

Turning the dried apple mango chips into juice involved grinding them into a fine powder first. Next, 10 g of this powder from each drying method (sun and oven) was mixed with 100 mm of purified water. The mixture was then given a good stir to make sure everything was evenly combined. To remove any leftover bits, the mixture was strained through a cheesecloth. Finally, the juice was tested to measure its sweetness (TSS [total soluble solids]), tartness (TA [titratable acidity]), and vitamin C content (ascorbic acid). This whole process was repeated for both types of dried apple mango chips to see how the drying method affected the quality and nutrients in the final juice.

### 2.5 Research design

The study was conducted using a completely randomized design due to the homogeneous laboratory conditions. Two factors were considered: the ripening stage and the drying method. The ripening stage had three levels: unripe, intermediate, and fully ripe. The drying method had two levels: oven‐drying and solar drying. These methods are well‐suited for Ethiopia, providing a balance between cost, availability, and functionality. Additionally, the drying temperatures utilized in our study were carefully chosen based on established recommendations from various researchers. This resulted in six treatment combinations (3 ripening stages × 2 drying methods). This gave us a total of six different ways we could treat the apple mangoes (3 ripening stages × 2 drying methods). To make sure the results were reliable, we repeated each treatment three times. In total, we produced 18 distinct batches of apple mango chips and juice, as detailed in Table 1. This experimental design provided a clear understanding of how both ripeness levels and drying methods influenced the quality of the dried apple mangoes and the resulting juice.

**TABLE 1:** Total experimental units with replications.

Factor one (ripening stage)	Factor two (drying method)					
	Oven dry (O)			Solar dry (S)		
R1 (unripe)	R1O	R1O	R1O	R1S	R1S	R1S
R2 (half ripe)	R2O	R2O	R2O	R2S	R2S	R2S
R3 (fully ripe)	R3O	R3O	R3O	R3S	R3S	R3S

### 2.6 Data were collected

#### 2.6.1 Physicochemical analysis

##### 2.6.1.1 Determination of proximate composition

The moisture, protein, fat, fiber, and ash content of the samples were determined by the method of the Association of Official Analytical Chemists (AOAC, 2000); the official method numbers are 962.09, 920.87, 925.09, 962.09, 923.03, respectively.

##### 2.6.1.2 pH and titratable acidity

The pH measurements were conducted following the Romanian Standard Methods 90/2007 using a Hanna digital pH meter. Five gram of the sample was weighed into a 50‐mL beaker, and 25 mL of distilled water was added. The mixture was stirred vigorously for 20 min and then allowed to stand for 30 min to let the suspended ions settle. The pH meter was calibrated using standard buffers at pH 7 and 4. The electrode was inserted into the partly settled suspension, and the pH value was read and recorded.

The TA of the apple mango juice was determined using the AOAC (2000) method. Five milliliters of the juice filtrate was diluted with distilled water to a total volume of 50 mL. From this solution, a 5 mL aliquot was taken and titrated with 0.1 N NaOH, using phenolphthalein as an indicator. The titration was continued until the orange color of the juice changed to pink. Triplicate measurements were performed, and the TA was calculated as a percentage of citric acid using the following equation:

$$\begin{array}{ccc} & & \mathrm{TA}\;\left(\%\right)\\ & & \;=\frac{\mathrm{mls}\;\mathrm{NaOH}\;\mathrm{used}\;\times \;0.1\;N\;\mathrm{NaOH}\;\times \;\mathrm{equivalent}\;\mathrm{factor}}{\mathrm{Volume}\;\mathrm{of}\;\mathrm{sample}}\\ & & \times 100 \end{array}$$

Given that citric acid is the dominant acid in apple mangoes, the mill equivalent factor for citric acid used in the calculations is 0.064.

##### 2.6.1.3 Total soluble solids

The concentration of dissolved sugars in the apple mango juice was determined using a refractometer, following the AOAC (2000) method. The refractometer was first calibrated with distilled water to ensure accuracy. Then, two drops of the juice sample were placed on the prism of the refractometer. Triplicate measurements were taken for each sample to ensure precision. The results were expressed in degrees Brix (°Brix), which represents the amount of total dissolved solids in 100 g of the juice product. This measurement indicates the sugar content of the juice, providing valuable information about its sweetness and overall quality.

##### 2.6.1.4 Ascorbic acid

###### 2.6.1.4.1 Determination of vitamin C (ascorbic acid)

The analysis of vitamin C in the sample was carried out using a titration method. Initially, 10 mL of the sample was mixed with an iodine solution until the endpoint was observed. To prepare the sample for titration, 10 mL of the extract was combined with 50 mL of distilled water in a volumetric flask. Furthermore, 10 drops of 1% starch solution were added and mixed well until the point of equivalence was attained. The endpoint of the titration was determined as the initial appearance of a dark blue‐black color due to the formation of the starch‐iodine complex, which persisted after swirling the solution for 20 s. The volume of iodine solution used during titration was noted by calculating the difference between the initial and final volumes. For the standardization of the titrant, 2.5 g of pure vitamin C was titrated against 40 mL of iodine solution to establish a reference for subsequent analyses. Ultimately, the concentration of ascorbic acid in the samples was calculated taking into account the dilution factor resulting from the sample and water volumes used, in conjunction with the standard iodine volume utilized during titration, based on the equation derived from the standardization process (where 2.5 g of vitamin C reacted with 40 mL of iodine solution) and dilution factor (DF) = total volume/sample volume. The final calculation for determining vitamin C content was expressed as follows: Vitamin C = (*Y* mL × 2.5 g/40 mL) × DF, where *Y* mL represents the volume of iodine solution required to achieve the endpoint during titration, and 40 mL denotes the amount used during the standardization with 2.5 g of vitamin C.

##### 2.6.1.5 Rehydration capacity

Rehydration experiments were conducted based on the method described by Tepe and Tepe (2020). Apple slices were arranged in a single layer of aluminum foil and dried in a forced‐air circulation oven. The rehydration process took place in a water bath at 50°C for 20 min. A 250‐mL glass container was used, containing 200 mL of water. During rehydration, the dried apple slices absorbed water, increasing mass. The rehydration ratio (RR) of the apple slices was calculated using the formula: $\mathrm{RR}=\frac{\mathrm{Mr}}{\mathrm{Mo}}\times 100$

##### 2.6.1.6 Color characteristics

The color of the dried apple mango chips flour was measured using a hand‐held spectrophotometer (KONICA MINOLTA.INC.). The Chroma meter was first calibrated with a white tile. The sample flour was poured to fill a Petri dish and then covered. The lens of the Chroma meter was placed on the Petri dish in three different parts. The color measurements were then taken and recorded as L* = darkness/lightness (0 = black, 100 = white), a* (−a = greenness and +a = redness), and b* (−b = blueness, +b = yellowness) (Greene & Bovell‐Benjamin, 2004).

### 2.7 Sensory analysis

Following the production of apple mango chips, a sensory evaluation was conducted with 50 panelists who were not trained in sensory analysis. These panelists, randomly selected from Injibara University staff and FSPT students, assessed the sensory attributes of the chips at different ripening stages. The attributes evaluated included taste, aroma, color, flavor, and overall acceptability. To ensure unbiased results, samples were randomly coded and presented in a controlled environment with consistent ambient temperature, uniform lighting, minimal noise, and good air circulation. Panelists used a five‐point hedonic scale to rate each attribute: 1 for “dislike it very much,” 2 for “dislike moderately,” 3 for “neither like nor dislike,” 4 for “like,” and 5 for “like very much.” Panelists were selected based on their health status to ensure they were familiar with the evaluation process and had no health issues that could affect their sensory perceptions. During the tasting sessions, each panelist evaluated one sample at a time and was instructed to cleanse their mouth with water between samples to minimize any residual flavors. This methodology was designed to enhance the accuracy and reliability of the sensory data collected.

### 2.8 Statistical analysis (methods of analysis)

All of the independent parameters of dried apple mango chips were tested in triplicate, and the findings were represented as means with standard deviations and analyzed using the Minitab version 19.2 software package. The statistical significance was examined by analysis of variance (ANOVA) using Minitab version 19.2 software package for each response, and the significance test level was set at 5% (*p* < 0.05). Additionally, the line graph and bar graph were analyzed using Sigma Plot software, version 15.

## 3 RESULTS AND DISCUSSION

### 3.1 Effects of ripening stages and drying methods on physicochemical properties of dried apple mango chips

**FIGURE 1:**
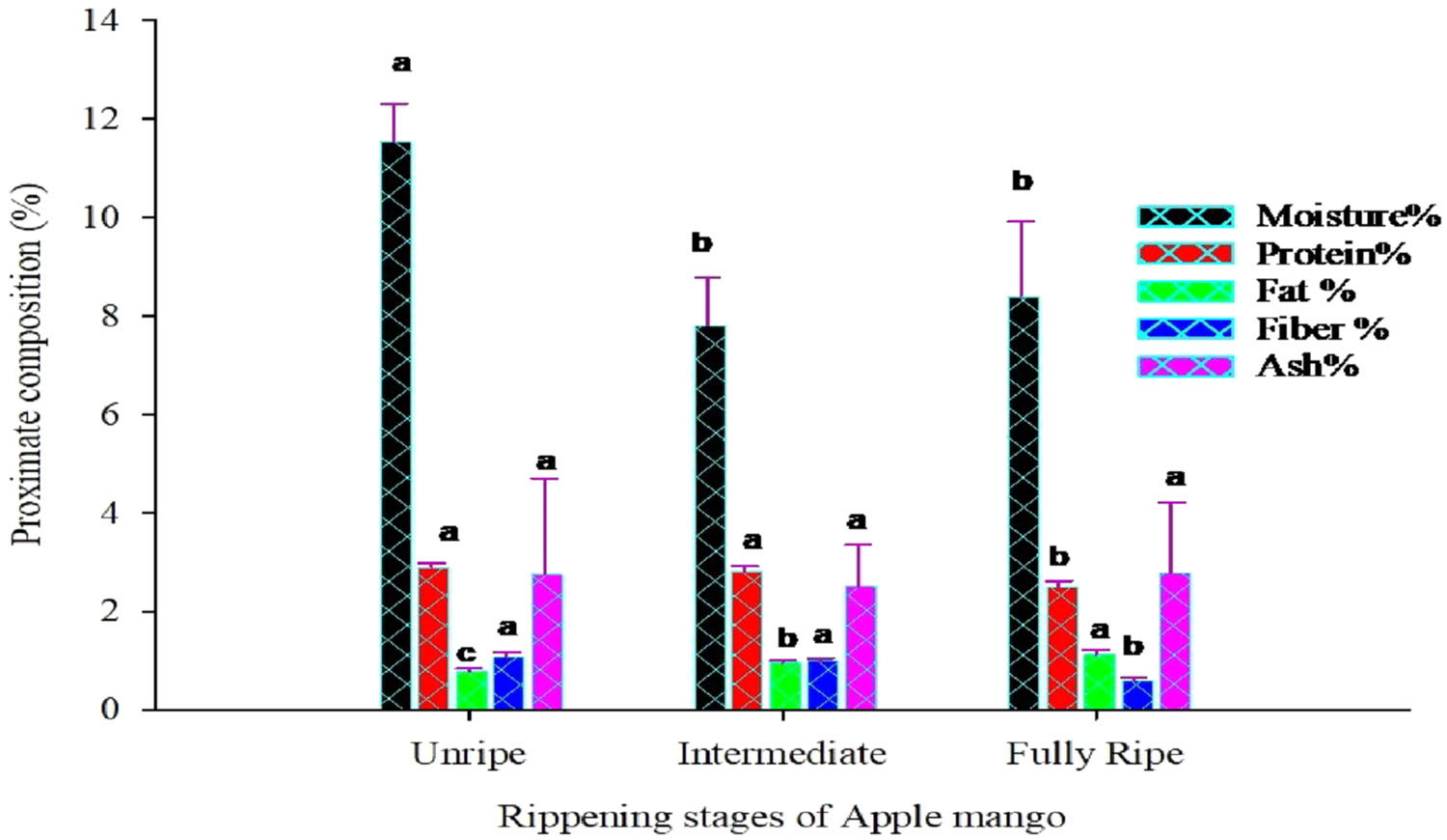
The mean separation letters (a, b, and c) indicate significant differences (p < 0.05) among the means, except for ash, where no significant difference was observed.

The physicochemical characteristics of dried mangoes were investigated and addressed in this study (Table 2). The ripening stages and drying methods of the mango had a significant impact on the physicochemical characteristics of dried apple mangoes. Such as moisture and protein content, L*, a*, pH, and vitamin C. It is interesting to note that only the ripening stages of the apple mango had an impact on the physicochemical properties of dried apple mangoes (*p* < 0.05) such as fat, fiber, b*, TA, TSS, and rehydration capacity (RC). However, the ripening stages of the apple mango did not significantly (*p* > 0.05) affect the ash content of the dried apple mango chips (Figure 1). Conversely, the drying methods did not have a significant (*p* < 0.05) effect on fat, fiber, b*, TA, TSS content, and RC of the dried chips.

**TABLE 2:** Result of physicochemical properties of mango chips as affected by the main effect (ripening stages and drying methods).

Factors		Moisture (%)	Protein (%)	Fat (%)	Fiber (%)	L*	a*	b*	pH	TA (g/100 g)	Ash (%)	TSS (°Brix)	Vitamin C (mg/100 g)	Rehydration capacity
Ripening stages	Unripe	11.53 ± 0.77a	2.89 ± 0.09a	0.78 ± 0.05c	1.06 ± 010a	59.02 ± 1.69c	11.45 ± 0.49c	49.12 ± 1.21b	4.05 ± 0.24c	0.90 ± 0.12a	2.44 ± 1.94a	25.70 ± 0.88c	25.60 ± 10.18a	52.14 ± 3.97b
	Intermediate	7.81 ± 0.97b	2.81 ± 0.10a	0.95 ± 0.05b	0.98 ± 0.06a	63.50 ± 1.33b	12.79 ± 0.11b	50.02 ± 0.17b	5.19 ± 0.23b	0.32 ± 0.08b	2.30 ± 0.84a	28.68 ± 1.23b	14.07 ± 0.08b	61.88 ± 1.33a
	Fully ripe	8.40 ± 1.51b	2.49 ± 0.12b	1.11 ± 0.10a	0.59 ± 0.07b	66.38 ± 3.43a	13.09 ± 0.11a	52.93 ± 0.05a	5.65 ± 0.88a	0.16 ± 0.04c	2.58 ± 1.42a	30.27 ± 0.54a	11.50 ± 1.73b	58.04 ± 2.80a
	***p*‐value**	**<0.05**	**<0.05**	**<0.05**	**<0.05**	**<0.05**	**0.000**	**0.000**	**0.000**	**0.000**	0.943	**0.000**	**0.000**	**0.001**
Drying methods	Solar	10.08 ± 1.56a	2.80 ± 0.17a	0.94 ± 0.16a	0.86 ± 0.20a	64.69 ± 4.04a	12.27 ± 0.97b	50.91 ± 1.73a	4.74 ± 0.48b	0.45 ± 0.37a	2.57 ± 1.51a	27.98 ± 2.24a	20.89 ± 10.33a	58.67 ± 6.73a
	Oven	8.41 ± 2.09b	2.65 ± 0.21b	0.95 ± 0.17a	0.90 ± 0.25a	61.25 ± 2.80b	12.61 ± 0.55a	50.46 ± 1.95a	5.19 ± 1.10a	0.47 ± 0.33a	2.38 ± 1.26a	28.46 ± 2.13a	13.23 ± 3.63b	56.04 ± 3.40a
	***p*‐value**	**<0.05**	**<0.05**	0.657	0.160	**<0.05**	**0.000**	0.183	**0.002**	0.565	0.439	0.157	**0.000**	0.106

*Note*: Means that do not share a letter are significantly different. Results are mean values of triplicate determination and mean with the different letters across the column are significantly different (*p* < 0.05).

Abbreviations: TA, titratable acidity; TSS, total soluble solids.

The moisture content of the dried apple mango chips was significantly (*p* < 0.01) affected by the main effects of ripening stages and drying methods (Table 2). In this case, the moisture content ranged from 7.81% to 11.50% in intermediate and unripe, respectively, while ranging from 8.41% to 10.08% in oven and solar drying methods, respectively (Figure 1). The data indicate that the moisture content in the apple mango chips was recorded using a solar drying method rather than an oven. The moisture content of dried apple mango in the current findings is consistent with Akther et al. (2021), who observed that the moisture content of dried apple mango powder using different dryers ranged from 5.80% to 12%. Similarly, the moisture content found in the current findings is consistent with Ampah et al. (2022), who observed that the moisture content of Kent, Keitt, Haden, and Palmer dried mangoes was found to be in the range of 6.72–10.61%, 5.60–11.87%, 8.50–11.20%, and 7.50–11.10%, respectively. However, the current findings are slightly lower than those reports, thus it is good for making different foods and can have a good shelf life of the products. This idea is supported by Tahmasebi et al. (2011), who said that as drying progresses and moisture content decreases, the dry matter content of the sample increases relative to the water content, which subsequently reduces the rate of moisture removal from the sample. While higher drying temperatures can accelerate moisture removal, prolonged exposure may lead to case hardening, which can impede further moisture loss (Dudko et al., 2022; Hamdan et al., 2021). The balance between temperature and airflow during drying is crucial, as increased gas flow can reduce diffusion barriers, thereby optimizing moisture removal rates (Dudko et al., 2022). The protein and fat content of apple mango chips varied based on ripening stages and drying methods. Specifically, the protein content ranged from 2.49% to 2.89%, while the fat content ranged from 0.78% to 1.11% (Table 2). The data indicate that as the ripening stages increased, the protein content in the apple mango chips decreased, whereas the fat content increased (Figure 1). Teshome (2010) reported protein content in dried apple mango leather ranging from 1.75% to 2.81%, and Osunde (2017) found protein content ranging from 2.9% to 4.64% in dried apple mango. Additionally, Dereje and Abera (2020b) found that the protein content values were between 2.49% and 2.71%. The study's findings that dried mango chips have protein and fat content within previously reported ranges have important implications for the food industry in Ethiopia and beyond. These results support accurate nutritional labeling, aid in product development and diversification, and help establish quality standards, all of which enhance export opportunities. By promoting dried mango chips as a value‐added, healthy snack, the Ethiopian food industry can boost the mango value chain, create economic opportunities, and expand its global market presence, while also educating consumers and encouraging healthier eating habits.

Examining the impact of drying methods, the mean protein content of apple mango chips varied from 2.65% to 2.80%. The lowest protein content was observed in chips dried using an oven, while the highest was observed in chips dried using solar drying. This indicates that solar drying has a significantly higher impact on retaining protein content compared to oven‐drying. This finding is consistent with Mwamba et al. (2017), who also observed similar protein content levels in mango dried in an oven (2.58%) compared to sun‐dried mango (1.66%). The study demonstrates that both the ripening stage and drying method significantly influence the protein and fat content of apple mango chips. Specifically, solar drying appears more effective in preserving protein content compared to oven‐drying. Research indicates that solar drying can retain up to 96.48% of protein content in dried products, such as country bean seeds, outperforming oven‐drying methods (Suborna et al., 2024). This advantage is attributed to the gentler drying conditions of solar drying, which typically operates at lower temperatures, minimizing thermal degradation of sensitive nutrients like proteins and vitamins (Ali et al., 2022). Although solar drying may require more time for heat absorption, its ambient temperature operation reduces the risk of nutrient loss, making it more efficient in nutrient preservation (Koşan et al., 2023). Moreover, using solar energy not only preserves nutrients but also supports sustainable agricultural practices, presenting an eco‐friendly option for smallholder farmers (Watson et al., 2024). In contrast, oven‐drying, while achieving faster moisture removal, often results in greater nutrient loss due to higher temperatures and prolonged exposure, emphasizing the need for selecting appropriate drying methods to ensure optimal nutrient retention.

**FIGURE 2:**
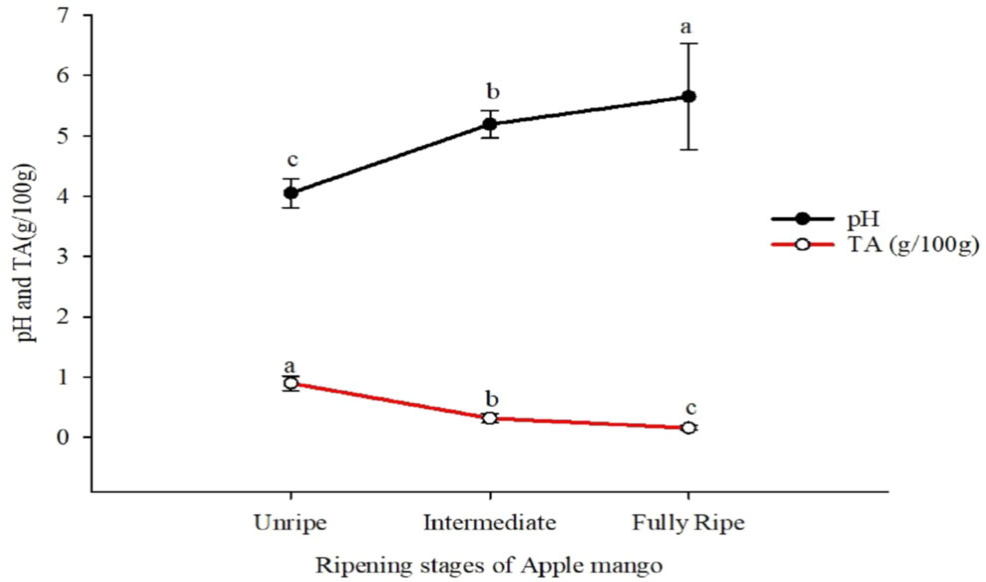
The mean separation letters (a, b, and c) indicate significant differences (p < 0.05) among the means.

The mean TA values ranged from 0.16 to 0.90/100 g across all ripening stages (Table 2), with the lowest observed in the fully ripe stage and the highest in unripe apple mango chips (Figure 2). This trend indicates a decrease in TA as apple mango ripening progresses, suggesting a reduction in sourness and potential enhancement of sweetness, consistent with previous findings of Dissa et al. (2011), who observed a significant decline in TA with ripening. The decreases in acidity suggest a reduction in sourness with the potential of improving the sweet taste as was observed in chips produced (Appiah et al., 2011). It is worth noting that Belay and Alemu (2020) reported a TA of 1.64/100 g in dried mango peel flour, which is higher than the values observed in our study. On the other hand, the pH values of dried apple mango chips ranged from 4.05 to 5.65 across ripening stages, with the lowest in unripe and the highest in fully ripe mangoes, with the lowest pH observed in the unripe stage and the highest in fully ripe apple mango chips (Figure 2). These pH levels are higher than those found by Dereje and Abera (2020), who reported pH ranges of 3.17–3.68 in mango slices. The higher pH is beneficial as it leads to a milder, less acidic taste, enhancing the sensory appeal and making the product suitable for various culinary uses. It also contributes to better preservation, potentially extending shelf life, and reflects the natural ripening process, resulting in a sweeter flavor.

**FIGURE 3:**
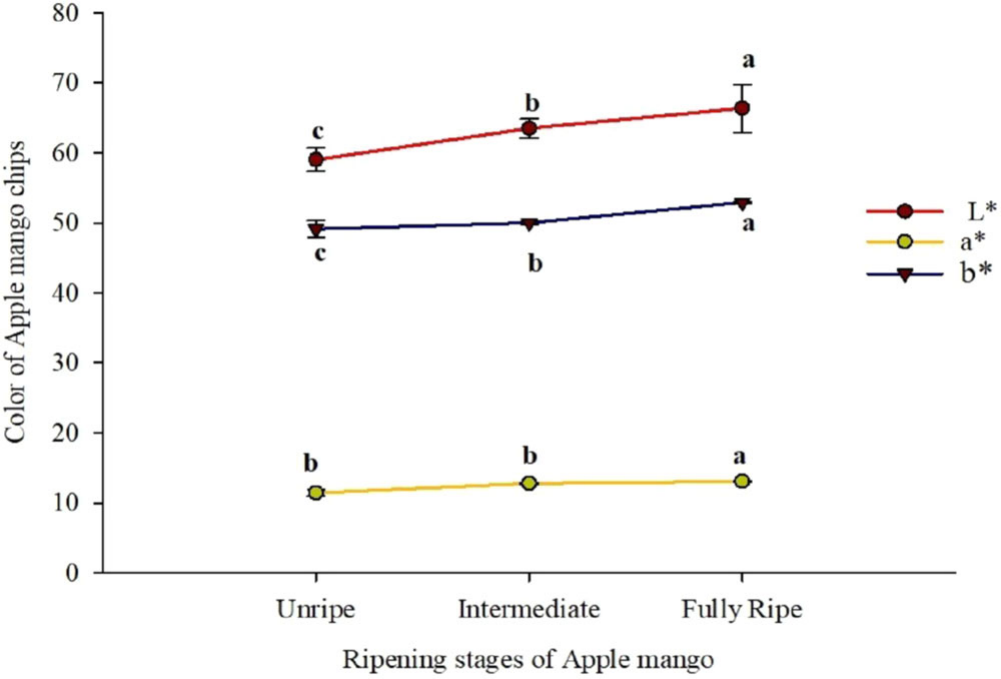
Figure 3.The mean separation letters (a, b, and c) indicate significant differences (p < 0.05) among the means.

The color (b*) value of dried apple mango chips significantly increases with ripening stages (*p* < 0.001), ranging from 49.12 for unripe to 52.93 for fully ripe apple mangoes (Table 2). The data indicate that the b* values in the apple mango chips were increased across ripening stages (Figure 3). These findings align with D. Li et al. (2023), who reported that b* values changed from 47.31 to 34.32 as apple mangoes matured. However, the current b* values are higher than those reported by Dereje and Abera (2020a), who found values ranging from 30.47 to 35.12 in dried apple mango samples, with significant differences among them. The increase in b* values is due to higher sugar concentration, increased carotenoid content, and decreased acidity in riper fruits, resulting in more yellow color. The increase in b* values as ripening stages advance is consistent with the observations of Deng et al. (2019). I. Nyangena, Owino, Ambuko et al. (2019) attributed this to better carotenoid retention, responsible for the typical yellow color of mangoes. High b* values result in more yellow products, which are preferred for dried mango products (Salehi & Kashaninejad, 2018). Additionally, research by Akoy (2014) and D. Li et al. (2023) indicates that b* values decrease with higher drying temperatures and longer drying times.

**FIGURE 4:**
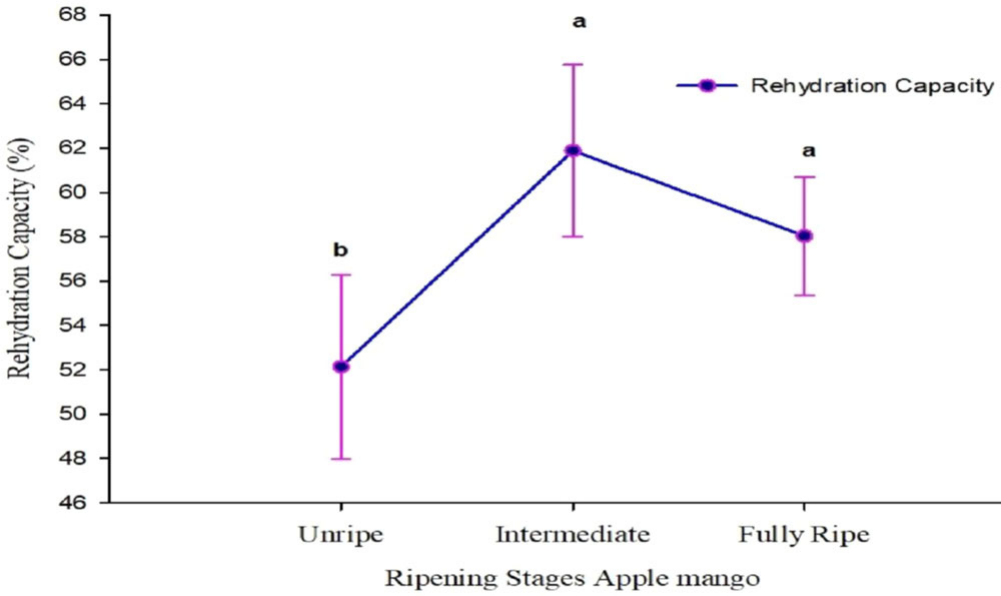
The mean separation letters (a and b) indicate significant differences (p < 0.05) among the means.

The RC of dried apple mango chips significantly increases with ripening stages (*p* < 0.001), ranging from 52.14% for unripe to 61.88% and 58.04% for intermediate and fully ripe apple mangoes, respectively. This trend, illustrated in Table 2 and Figure 4, shows an increase in RC across ripening stages. These findings are lower than those reported by Salehi, Inanloodoghouz, Ghazvineh et al. (2023), who observed rehydration capacities ranging from 127.27% to 137.07% in dried sweet cherries. However, the values for dried apple mango chips are higher than those reported by Suherman et al. (2023), where an RC of 2.852% was noted in freeze‐dried mango slices with a moisture content of 6.574%. The higher RC of dried mango enhances its texture, flavor, and nutritional value, making it more appealing and versatile for various culinary uses. This quality allows the mango to retain more moisture, resulting in a product that closely resembles fresh fruit when rehydrated. Consumers prefer such products for their improved eating experience, and they offer extended shelf life and storage benefits, making them a valuable option for both consumers and manufacturers. The RC of dried mango varies significantly based on the drying method employed and the specific characteristics of the mango used.

### 3.2 Effects of ripening stages and drying methods interaction on physicochemical properties of dried apple mango chips

The proximate and physicochemical characteristics of dried apple mango chips were investigated and addressed in this study (Table 3). The interaction between ripening stages and drying methods had a significant (*p* < 0.05) impact on some proximate and physicochemical characteristics of dried apple mango chips. However, the interaction between ripening stages and drying methods did not have a significant (*p* > 0.05) effect on the protein, fat, moisture, and ash content, TA, and RC.

**TABLE 3:** Result of physicochemical properties of dried mango chips as affected by the interaction of ripening stages and drying methods.

Factors	Moisture (%)	Protein	Fat	Fiber	L*	a*	b*	pH	TA (g/100 g)	Ash (%)	TSS (°Brix)	Vitamin C (mg/100 g)	Rehydration capacity
Unripe—Oven	11.12 ± 0.97ab	2.81 ± 0.29ab	0.79 ± 0.04b	1.15 ± 0.03a	57.96 ± 1.94d	11.89 ± c	48.54 ± 0.51b	3.92 ± 0.04c	0.87 ± 0.14a	2.30 ± 1.97a	25.73 ± 1.10d	m 16.90 ± 4.19b	52.88 ± 1.76b
Unripe—Solar	11.93 ± 0.19a	2.97 ± 0.03a	0.76 ± 0.07b	0.97 ± 0.04b	60.09 ± 0.07cd	11.00 ± d	49.69 ± 1.56b	4.18 ± 0.31c	0.92 ± 0.12a	2.50 ± 2.22a	25.67 ± 0.84c	34.29 ± 3.86a	51.39 ± 5.89b
Intermediate—Oven	6.98 ± 0.03e	2.77 ± 0.14bc	0.95 ± 0.07ab	0.96 ± 0.04b	62.38 ± 0.54bc	12.86 ± 0.12ab	49.94 ± 0.14b	5.23 ± 0.26b	0.39 ± 0.03b	2.04 ± 0.98a	29.73 ± 0.46a	12.61 ± 0.55bc	58.82 ± 2.59ab
Intermediate—Solar	8.63 ± 0.05cd	2.84 ± 0.06ab	0.95 ± 0.05ab	0.99 ± 0.08ab	64.63 ± 0.55b	12.71 ± 0.04b	50.09 ± 0.20b	5.15 ± 0.27b	0.25 ± 0.05bc	2.36 ± 0.44a	27.63 ± 0.49b	15.53 ± 0.40bc	64.93 ± 2.26ab
Fully ripe—Oven	7.12 ± 0.12de	2.39 ± 0.04d	1.13 ± 0.13a	0.59 ± 0.07c	63.40 ± 1.50b	13.07 ± 0.12a	52.90 ± 0.03a	6.42 ± 0.36a	0.15 ± 0.05c	2.21 ± 1.06a	29.90 ± 0.17a	10.17 ± 0.15c	56.41 ± 3.13ab
Fully ripe—Solar	9.68 ± 0.91bc	2.59 ± 0.04c	1.10 ± 0.09a	0.60 ± 0.08c	69.36 ± 0.66a	13.10 ± 0.11a	52.96 ± 0.06a	4.88 ± 0.03b	0.17 ± 0.02bc	2.27 ± 1.98a	30.63 ± 0.55a	12.83 ± 1.47bc	59.68 ± 1.32ab
***p*‐value**	0.076	0.253	0.926	**<0.05**	**<0.05**	**<0.05**	**0.335**	**<0.05**	0.159	0.837	**<0.05**	**<0.05**	0.157

*Note*: Means that do not share a letter are significantly different. Results are mean values of triplicate determination and mean with the different letters across the column are significantly different (*p* < 0.05).

Abbreviations: TA, titratable acidity; TSS, total soluble solids.

#### 3.2.1 Fiber

The fiber content of apple mango chips showed significant variation depending on the drying method used. The mean fiber content ranged from 0.59% to 1.15% (Table 3). The lowest fiber content was observed in fully ripe apple mangoes dried using an oven, while the highest fiber content was found in unripe apple mangoes also dried using an oven. These values are higher than the average fiber content influenced solely by ripening stages (Table 2). The data suggest that as apple mangoes progress through different ripening stages, the fiber content in the apple mango chips increases. A study by Ibarra‐Garza et al. (2015) found that total dietary fiber content in mangoes increased during the early ripening stage. Another study by Joshi et al. (2023) observed that the maximum levels of crude fiber were reached on the 12th day of storage in mangoes harvested at 7–9 °Brix TSS and 9–11 °Brix TSS, indicating a rise in fiber content as the mangoes ripened. However, Ibarra‐Garza et al. (2015) also noted that at the final ripening stage, the total dietary fiber content diminished by 13% compared to the initial stage. The higher fiber content in unripe apple mangoes dried using solar drying can be attributed to the slower and more gentle drying process of solar drying, which helps preserve the structural integrity and fibrous components of the apple mangoes better than the more intense oven‐drying process. When comparing these findings to previous studies, the fiber content values in this study are consistent with the results reported by Akther et al. (2020), who found fiber content in dried mangoes ranging from 0.85% to 3.75%. This indicates that the fiber content of mango chips, as influenced by different drying methods and ripening stages, falls within the range established by previous research.

#### 3.2.2 pH

The pH levels of dried apple mango chips, ranging from 3.92 to 6.42 (Table 3), were significantly influenced by the interaction between ripening stages and drying methods. Unripe oven‐dried samples had the lowest pH value of 3.92, while fully ripe oven‐dried samples had higher pH values compared to solar‐dried ones, indicating lower acidity and increased sourness at 10:16 a.m.

As mangoes ripened, the pH increased and then declined in acidity due to the maturity of the fruit and drying methods. This aligns with Ampah et al. (2022), who observed higher pH levels in dried mango samples. Oven‐drying elevates pH levels in dried mango chips through several mechanisms: consistently high temperatures trigger non‐enzymatic browning reactions like the Maillard reaction and caramelization, generating alkaline compounds. This process also degrades organic acids, reduces their concentration, and halts enzymes that typically produce acidic compounds. Additionally, rapid evaporation of volatile acids and water removal concentrates non‐volatile alkaline substances, further raising pH levels. These findings are consistent with Dereje and Abera (2020b), who noted that pH increases during ripening are due to acid utilization in respiration and reduced moisture content during drying. Compared to studies by Tasie et al. (2020) on different mango varieties (ranging from 3.86 to 4.73) and Mwamba et al. (2017) on dried mangoes (ranging from 1.65 to 2.65), the pH values in this research were higher, likely influenced by variations in mango varieties, ripening stages, and drying methods. These differences were also noted by Ampah et al. (2022), who attributed variations in dried mangoes to different mango varieties and drying techniques. The pH values recorded in this study were also higher than those reported by Dereje and Abera (2020b) (3.68–3.17) and Mwamba et al. (2017) (3.09–4.11) for dried mangoes, further emphasizing the impact of drying methods on pH levels. Higher pH values in dried mango chips can enhance safety by inhibiting spoilage microorganisms, extending shelf life, and creating a milder, less acidic taste that appeals to consumers. Additionally, a higher pH can improve texture and help preserve nutrients sensitive to acidic conditions, thereby maintaining the overall quality of the dried mango chips.

#### 3.2.3 Total soluble solids (TSS)

The TSS of dried apple mangoes are significantly influenced by the interaction between drying methods and ripening stages (*p* ≤ 0.01), with values ranging from 25.67 to 30.63 °Brix (Table 3). The lowest TSS value (25.67 °Brix) was observed in unripe apple mangoes dried using solar methods, while the highest value (30.63 °Brix) was found in fully ripe apple mangoes dried by the same method. The increase in TSS in fully ripe apple mangoes dried using solar methods can be attributed to the fruit's maturity. The current values of TSS are higher compared to those reported by Okoth et al. (2013), who found that Kent varieties had lower TSS levels at 6.50 °Brix across all stages. These higher values are significant as they indicate an increase in sugar content or overall quality. Ripening significantly influences total solubility, with observed increases during this process (Appiah et al., 2011). The sucrose content of mangoes has been noted to increase during ripening, contributing to the rise in TSS (Belay & Alemu, 2020).

Furthermore, the drying process also influences TSS levels due to the reduction in moisture content, leading to a higher percentage of TSS and impacting the flavor profile of dried mangoes (Dereje & Abera, 2020a). Changes in cell wall structure and the breakdown of complex carbohydrates into simple sugars also play a role in increasing TSS levels during drying (Z, 2016). Selecting an appropriate drying method is crucial to avoid undesirable changes and maintain the quality of the dried product (Y. Li et al., 2019).

Various reports have highlighted the impact of drying methods on TSS levels, with discrepancies noted in maximum TSS values. For example, a reported value of 8.18 °Brix was obtained through solar drying (El‐beltagi et al., 2023), whereas the current findings are higher. An increase in TSS occurs as moisture content reduces during drying, which leads to solid dilution (El‐beltagi et al., 2023). The sweetness of fruit increases with higher TSS levels as starches are sweeter than glucose and fructose (Khan et al., 2020; Z, 2016). TSS is essential for the processing, storage, and quality of the food produced (Abe‐Inge et al., 2018), although the current findings may be lower compared to previous reports.

Other studies have reported TSS values ranging from 22.36 to 52.02 °Brix for solar‐ and oven‐dried mangoes (Mwamba et al., 2017), and the current findings fall within this range. Variations in TSS levels among mango varieties have been documented, with differences attributed to drying methods and ripening stages (Tasie et al., 2020). For instance, Kent mangoes exhibited the highest TSS, followed by Apple, while Keitt had the lowest TSS (Tasie et al., 2020). TSS serves as an indicator of fruit maturity and is crucial for determining the harvesting time.

#### 3.2.4 Ascorbic acid (v C)

The mean values ranged from 10.17 to 34.29 mg/100 g, which was significantly (*p* < 0.01) affected by the interaction between ripening stages and drying methods as shown in Table 3. The lowest value (10.17 mg/100 g) was observed in fully ripe dried in the oven whereas the highest value was observed in unripe apple mango dried in solar method. The data highlight a significant decrease in vitamin C content in dried samples as apple mango ripening increased and interacted with the oven‐drying method. These effects include increased enzymatic activity, higher respiration rates, thermal degradation, prolonged heat exposure, and oxidative conditions. Variations between the observed and reported values in this study could stem from differences in ripeness stage, variety, geographical location, and post‐harvest practices (I. O. Nyangena, Owino, Imathiu, et al., 2019). The ANOVA also shows that oven‐dried apple mango samples had a significantly lower vitamin C content than solar‐dried samples. This reduction is attributed to heat recirculation within the heating chamber. Vitamin C content decreased with increasing drying temperatures from 50 to 65°C, with the lowest value (28.45 mg/100 g) observed in Ngowe variety samples oven‐dried at 65°C for 10 h (I. O. Nyangena, Owino, Imathiu, et al., 2019). Our findings align with previous studies, with El‐beltagi et al. (2023) reporting maximum ascorbic acid content in solar‐dried samples (23.18 mg/100 g) followed by oven‐dried ones (21.74 mg/100 g). The highest ascorbic acid values obtained in this study were also under the range values of those reported for dried samples by various methods (33.18–41.24 mg/100 g) (Dereje & Abera, 2020a). This observation is consistent with the heat sensitivity of vitamin C, as noted by Abrol et al. (2014). Additionally, vitamin C losses during drying depend on factors such as drying method, raw material type, and pre‐treatments (Wijewardana et al., 2016).

Teshome (2010) reported that the ascorbic acid values ranged from 14.98 to 44.44 mg/100 g for dried mango leather, which fall within our observed range and values reported by Dereje and Abera (2020b) (32.05–34.38 mg/100 g). Mohamed et al. (2017) reported a reduction in ascorbic acid content (24.29–71.88%) for dried mango, consistent with our findings. Solar drying appears to be a viable method, yielding satisfactory results compared to temperature‐controlled oven‐drying (Mwamba & Mputu, 2022), and it enhances several nutrients in fresh mangoes, including vitamins, minerals, and proteins.

#### 3.2.5 Color (L*)

The mean values ranged from 57.96 to 69.36, which was significantly (*p* < 0.05) affected by the interaction between ripening stages and drying methods as shown in Table 3. The lowest value (57.96) was observed in unripe apple mango dried in the oven, whereas the highest value was observed in fully ripe apple mango dried in the solar method. The data highlight a significant increase in L* value as apple mango ripening increased and interacted with the solar drying method. This increment may be due to the maturity of the fruits. The color L* value obtained in this study is in line with D. Li et al. (2023), who state that, as the maturity increased, the L* values changed from 71.92 to 60.35. However, the L* values of dried mango are slightly higher than 55.58, 55.28, 56.0, and 57.00 for slices of solar, tray, freeze, and fluidized bed, respectively (D. Li et al., 2023). The significant increase in L* value with ripening and solar drying is due to the combined effects of pigment changes during ripening and the gentle, uniform drying process of solar drying, which helps preserve the natural lightness of the mango slices.

#### 3.2.6 Color (a*)

The mean value of color a* ranged from 11 to 13.10, showing significant differences across ripening stages and drying methods. The lowest value for dried apple mango chips was observed in unripe fruit dried using solar drying, whereas the highest value was found in fully ripe apple mango dried using the same method, followed by 13.07 for fully ripe fruit dried in an oven. Fully ripe apple mangoes dried using solar drying have the highest a* values because the gradual and mild drying process preserves or enhances the carotenoid pigments more effectively than the controlled but higher temperature environment of oven‐drying, which may cause some pigment degradation. This is consistent with findings by D. Li et al. (2023), who reported a* color coordinate values of 12.62 for solar drying and 12.84 for tray drying, noting that color a* values increased with ripening stages, indicating redder and more intense coloration. Furthermore, Joshi et al. (2023) found that the physicochemical properties of apple mangoes, such as TSS and TA, varied with ripening stages, influencing color development during drying. This progression in color intensity and change in a* values as ripening advances highlights the impact of maturity status on the visual attributes of dried apple mango products, emphasizing the importance of considering ripeness when assessing color changes in dried apple mangoes.

### 3.3 Effects of ripening stages and drying methods on sensory properties of apple mango chips

The ripening stages of apple mangoes significantly impacted the sensory properties of dried apple mango chips (*p* < 0.01). Across ripening stages—unripe, intermediate, and fully ripe—the sensory attributes of dried apple mango chips, including color, taste, flavor, aroma, and overall acceptability, were notably influenced. As the apple mango ripened, there was an evident increase in the sensory acceptability of the chips, indicating a preference for sensory properties as ripeness progressed. The highest levels of all sensory attributes were consistently observed in the fully ripe stage. Additionally, the choice of drying method significantly affected the sensory properties of dried apple mango chips (*p* < 0.01). The solar drying method consistently yielded higher ratings for all sensory attributes compared to the oven‐drying method (Table 4). This suggests a higher preference for sensory properties in apple mango chips dried using the solar method over those dried in an oven.

**TABLE 4:** Result of physicochemical properties of mango chips as affected by the ripening stages and drying methods.

Factors		Color	Aroma	Taste	Flavor	Overall acceptability
Ripening stages	Unripe	2.06 ± 0.25c	1.83 ± 0.09c	1.94 ± c	1.80 ± 0.19c	1.83 ± 0.14c
	Intermediate	3.20 ± 0.26b	2.64 ± 0.36b	3.41 ± 0.22b	3.03 ± 0.39b	3.03 ± 0.28b
	Fully ripe	4.51 ± 0.52a	4.15 ± 0.67a	4.46 ± 0.50a	4.17 ± 0.61a	4.36 ± 0.43a
	***p*‐value**	**<0.05**	**<0.05**	**<0.05**	**<0.05**	**<0.05**
Drying methods	Solar	3.57 ± 1.16a	3.15 ± 1.35a	3.52 ± 1.22a	3.30 ± 1.33a	3.27 ± 1.33a
	Oven	2.94 ± 0.98b	2.59 ± 0.79b	3.03 ± 2.00b	2.71 ± 0.74b	2.88 ± 0.87b
	***p*‐value**	**<0.05**	**<0.05**	**<0.05**	**<0.05**	**<0.05**

### 3.4 Effects of the interaction between ripening stages and drying methods on sensory properties of apple mango chips

The sensory properties of dried apple mango chips were investigated and addressed in this study (Table 5). The interaction between ripening stages and drying methods had a significant (*p* < 0.05) impact on the sensory properties of dried apple mango chips.

**TABLE 5:** Result of physicochemical properties of mango chips as affected by the interaction between the ripening stages and drying methods.

Factors	Color	Taste	Flavor	Aroma	Overall acceptability
Unripe—Oven	1.88 ± 0.10e	1.85 ± 0.14d	1.85 ± 0.21d	1.86 ± 0.11d	1.90 ± 0.16e
Unripe—Solar	2.23 ± 0.23e	2.02 ± 0.06d	1.75 ± 0.03d	1.80 ± 0.07d	1.76 ± 0.01e
Intermediate—Oven	2.82 ± 0.14d	3.09 ± 0.10c	2.73 ± 0.07c	2.46 ± 0.61cd	2.84 ± 0.02d
Intermediate—Solar	3.57 ± 0.23c	3.73 ± 0.25b	3.33 ± 0.35b	2.81 ± 0.14bc	3.22 ± 0.22c
Fully ripe—Oven	4.12 ± 0.16b	4.13 ± 0.24b	3.53 ± 0.22b	3.46 ± 0.42b	3.90 ± 0.10b
Fully ripe—Solar	4.89 ± 0.06a	4.79 ± 0.06a	4.80 ± 0.08a	4.85 ± 0.03a	4.82 ± 0.12a
***p*‐value**	0.081	**<0.05**	**<0.05**	**<0.05**	**<0.05**

#### 3.4.1 Color

**FIGURE 5:**
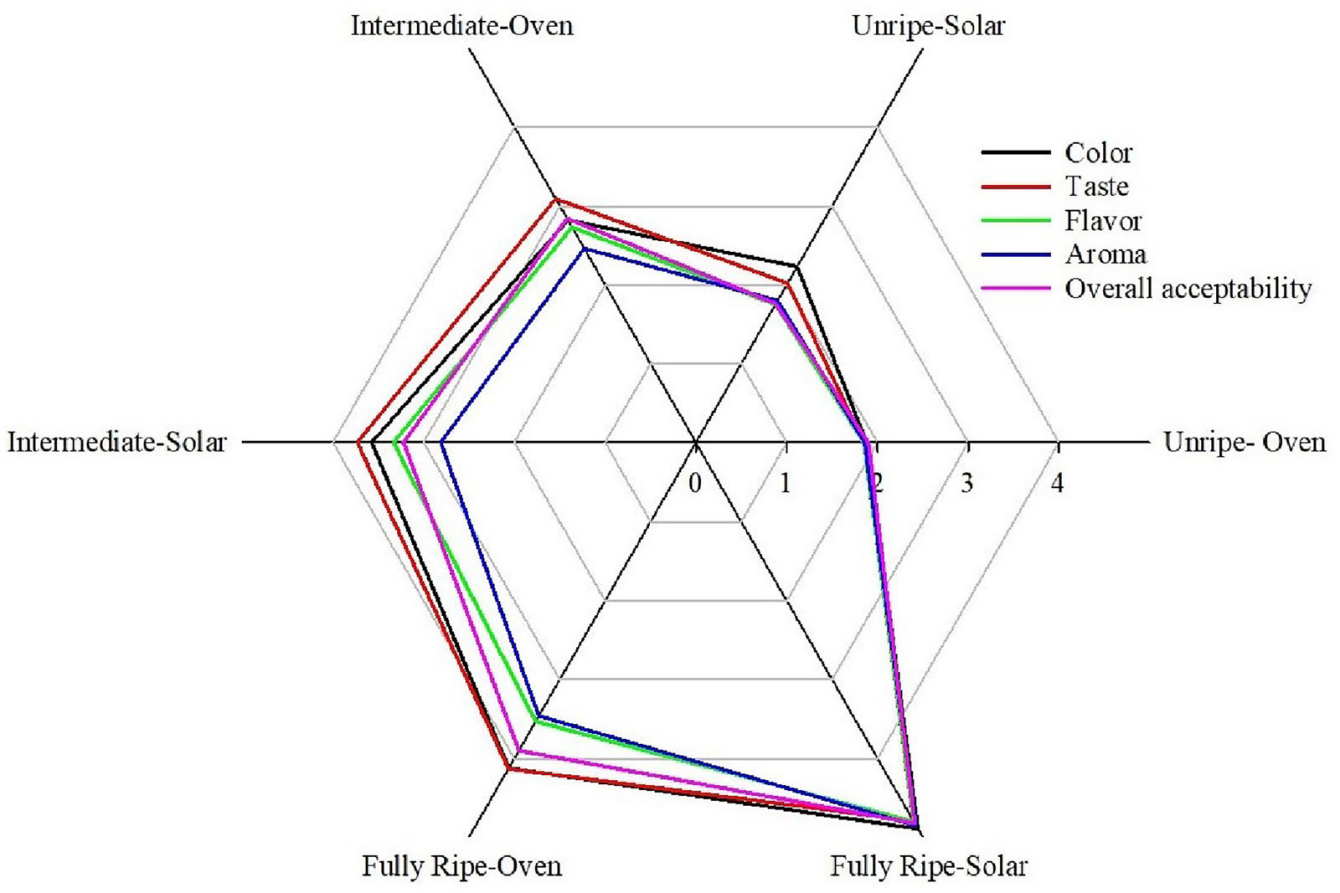
Effects of the interaction between ripening stages and drying methods on sensory properties of mango chips.

The color of apple mango fruit chips did not show a significant interaction effect (*p* > 0.05), with mean color values ranging from 1.88 to 4.89 (Table 5). The lowest mean value (1.88) was observed in chips made from unripe apple mangoes dried in an oven. In contrast, chips produced from fully ripe, solar‐dried apple mangoes scored the highest (4.89) in terms of color (Figure 5). Fully ripe mangoes generally yield better color quality in dried chips compared to those at unripe or intermediate stages. This is because fully ripe mangoes have higher sugar content and more developed pigments, which enhance color retention during drying (Mugodo & Workneh, 2021). Additionally, solar drying methods, particularly improved solar drying technologies, tend to preserve better color quality in dried mango chips than traditional oven‐drying. This is attributed to the gentler drying process and better control of drying conditions in solar dryers (Mohammed et al., 2020; Mugodo & Workneh, 2021).

#### 3.4.2 Taste

The taste of apple mango chips varied significantly based on the ripening stage and drying method (*p* < 0.05) (Table 5). Taste scores ranged from 1.85 to 4.79, with the lowest score (1.85) for chips from unripe apple mangoes dried in an oven. In contrast, chips from fully ripe apple mangoes dried using solar methods received the highest preference (4.79). Solar‐dried apple mango chips consistently exhibited superior taste profiles compared to oven‐dried chips across all ripening stages (Figure 5). Improved solar drying methods maintain better sensory qualities, including taste, aroma, color, and overall acceptability, compared to traditional solar drying and oven‐drying (Mohammed et al., 2020; Techane, 2022). Fully ripe mangoes dried with solar methods not only show higher RC and better physicochemical properties but also achieve better taste profiles (Techane, 2022). Ripe chips are particularly favored due to lower acidity and higher TSS (sweetness), which positively impact taste and pH. Conversely, higher TA correlates with lower taste preference. The improved taste in ripe chips is associated with increased sweetness and reduced acidity, as starches convert to sugars during ripening. Unripe chips, with higher organic acid levels, are less preferred due to their sour taste, highlighting the crucial role of sugar and acidity levels in determining the taste quality of apple mango chips.

#### 3.4.3 Aroma

The aroma of apple mango chips exhibited a significant variation (*p* ≤ 0.01), with mean values ranging from 1.80 to 4.85 (Table 5). The lowest aroma score (1.80) was recorded in chips made from unripe apple mangoes dried using solar methods, whereas chips made from fully ripe apple mangoes dried using solar methods achieved the highest score (4.85) (Figure 5). These fully ripe chips were preferred over those made from half‐ripe and unripe mangoes, regardless of whether they were dried by solar or oven methods. This enhancement in aroma in fully ripe apple mango chips can be attributed to the natural ripening process, during which the fruit develops a more complex bouquet of volatile compounds. These compounds, including esters, aldehydes, and terpenes, contribute to a richer and more appealing aroma. As mangoes ripen, their chemical composition undergoes significant changes, leading to a more pronounced and pleasant fragrance that enhances the overall sensory experience. This heightened aroma quality explains why panelists preferred the fully ripe solar‐dried mango chips over those made from less ripe fruit. The superior aroma not only indicates a more developed flavor profile but also reflects the fruit's optimal stage of ripeness, further underscoring the importance of ripeness in determining the sensory attributes of dried mango chips. Similar trends have been observed in other studies, such as the research by Mohammed et al. (2020) on the solar drying of pineapple slices. In this study, 3‐mm thick pineapple slices with a 2.5 cm diameter were dried using both an improved solar drying method at 40.3°C and traditional solar drying techniques. The findings revealed that the improved solar drying method significantly enhanced the sensory qualities of the pineapple slices, particularly the aroma, surpassing the performance of conventional solar drying methods. This international comparison highlights the critical role of optimized drying techniques and ripeness in enhancing the sensory qualities of dried fruit products.

#### 3.4.4 Flavor

The flavor of apple mango chips was significantly influenced by the interaction between ripening stages and drying methods (*p* < 0.01) (Table 5). Flavor, which includes the combined sensations perceived through both taste and smell, varied notably across different chip types. Chips made from fully ripe apple mangoes dried using solar methods were the most favored for their flavor, scoring 4.80. In contrast, chips from fully ripe mangoes dried in an oven had a slightly lower score of 3.53, while those made from intermediate‐ripeness fruit dried with solar methods were least preferred, scoring 3.33 (Figure 5). The lower flavor intensity observed in oven‐dried chips compared to solar‐dried ones can be attributed to the degradation of sugars and the loss of key volatile compounds that contribute to both aroma and flavor. The sensory panel's preference for fully ripe chips is likely due to their higher sugar content and balanced acidity, where the TSS positively correlated with mouthfeel satisfaction, while acidity levels inversely affected flavor perception. These factors emphasize the critical role of sugar content and acidity in shaping the flavor profile of apple mango chips. This trend mirrors findings from other studies, such as the research by Mohammed et al. (2020) on the solar drying of pineapple slices. In that study, pineapple slices (3 mm thick, 2.5 cm in diameter) were dried using both an improved solar drying method at 40.3°C and traditional solar drying techniques. The results showed that the improved solar drying method significantly enhanced the flavor and overall sensory qualities of the pineapple slices, outperforming conventional drying methods. This cross‐comparison highlights the impact of drying methods on the retention of flavor and the importance of optimizing these methods to enhance the sensory properties of dried fruit products, including mango and pineapple.

#### 3.4.5 Overall acceptability

Table 5 shows that the overall acceptability of apple mango chips was significantly affected (*p* < 0.01) by the interaction between the ripening stage and the drying method. Panelists consistently favored chips made from fully ripe apple mangoes, which received the highest overall acceptability score of 4.82, as depicted in Figure 5. This strong preference can be attributed to the higher TSS content in fully ripe mangoes, which results in a sweeter taste and more robust flavor profile. Regression analysis confirmed that taste was the most significant factor contributing to overall acceptability, emphasizing its crucial role in determining the sensory appeal of apple mango chips. The findings suggest that fully ripe mangoes, with their higher sugar content and enhanced flavor, are ideal for producing chips that align with consumer preferences in terms of taste and overall quality. These results underscore the importance of both the ripening stage and drying method in shaping the sensory characteristics of dried fruit products. By carefully optimizing these factors, producers can significantly enhance the overall acceptability of dried mango chips, meeting consumer expectations for taste, flavor, and satisfaction. This highlights the necessity of selecting the appropriate ripening stage and employing effective drying methods to maximize the sensory appeal and market potential of dried fruit products. This observation aligns with findings from other studies, such as the research by Mohammed et al. (2020) on the solar drying of pineapple slices. In that study, pineapple slices (3 mm thick, 2.5 cm in diameter) were dried using both an improved solar drying method at 40.3°C and traditional solar drying techniques. The improved method significantly enhanced the flavor and overall sensory qualities of the pineapple slices, outperforming conventional drying methods. This comparison further illustrates the critical impact of drying techniques on the overall acceptability of dried fruit products and the importance of optimizing these methods to achieve superior sensory properties.

### 3.5 Principal component analyses

**FIGURE 6:**
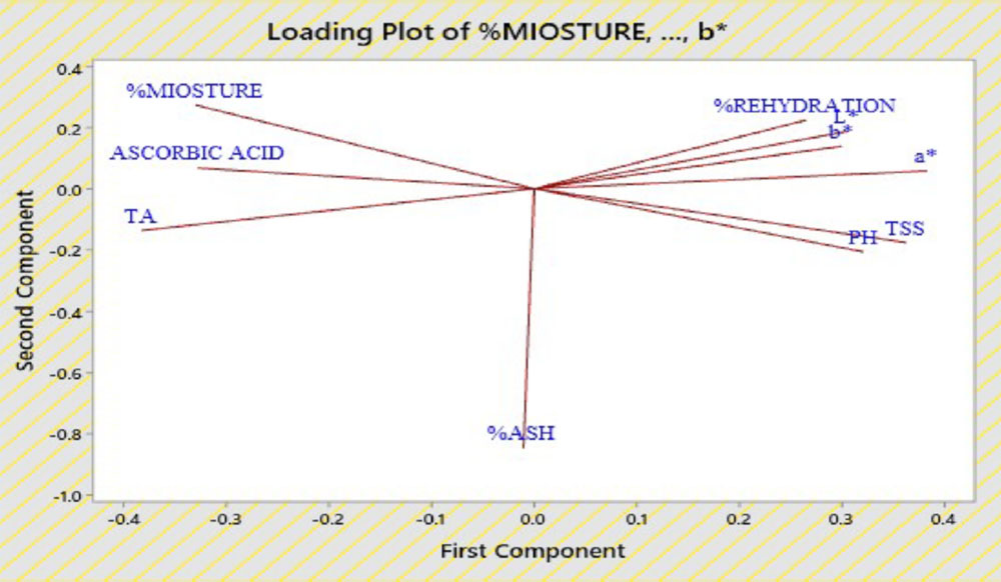
Loading plot of dried‐apple chips, illustrating the relationship between physicochemical properties. TA, titratable acidity; TSS, total soluble solids.

The principal component analysis (PCA) loading plot of dried apple chips, as shown in Figure 6, highlights the relationships between various physicochemical properties. In the plot, the length of each vector indicates the importance of the corresponding variable, with longer vectors exerting a stronger influence on the principal components. Positive correlations are observed among variables such as rehydration, color parameters (b*, L*, a*), TSS, and pH, as these vectors are closely aligned. Conversely, moisture, ascorbic acid, and TA show negative correlations with the aforementioned variables, as evidenced by their opposing vectors. The first principal component (x‐axis) captures the most significant variance, with rehydration, b*, L*, a*, TSS, and pH contributing prominently. The second principal component (y‐axis) emphasizes the role of moisture, ascorbic acid, and TA. Additionally, percentage ash stands somewhat apart, indicating it has a unique contribution that is not strongly correlated with the other variables. Overall, the PCA loading plot reveals an inverse relationship between moisture, ascorbic acid, TA, and other physicochemical properties, offering valuable insights for optimizing the drying process and improving the quality of dried apple chips.

## 4 CONCLUSION

This study highlights the significant influence of ripening stages and drying methods on the quality of dried apple mango chips. The findings demonstrate that solar drying, especially when applied to fully ripe mangoes, produces chips with superior physicochemical and sensory attributes compared to oven‐drying. Specifically, solar‐dried chips exhibited better color quality, higher moisture retention (7.81%–11.50%), and higher TSS (25.67–30.63 °Brix). The fully ripe, solar‐dried chips were also preferred in sensory evaluations, scoring the highest in terms of color, taste, flavor, aroma, and overall acceptability. In contrast, oven‐dried chips, particularly those from fully ripe mangoes, had higher pH values (3.92–6.42) and the lowest vitamin C content (10.17 mg/100 g), indicating a potential loss of some nutritional qualities during the drying process. These results underscore the importance of selecting the appropriate ripening stage and drying method to optimize the quality of dried mango products. The study provides valuable insights for the food industry, particularly in regions where advanced drying technologies like freeze‐drying or vacuum drying are not readily accessible. By focusing on solar drying, which is both cost‐effective and environmentally friendly, producers can enhance the marketability of dried mango chips. However, the study has some limitations. The reliance on solar drying and oven‐drying methods may limit the applicability of the findings to contexts where more advanced drying technologies are available. Additionally, the exclusive focus on apple mangoes means the results may not be fully generalizable to other mango varieties or fruits. The sensory analysis was conducted with a small, non‐trained panel, which could affect the robustness of the sensory data. Future research should explore the impact of advanced drying techniques, such as freeze‐drying and vacuum drying, on the quality of apple mango chips to provide a broader comparison. Expanding the study to include other mango varieties and fruits would offer a more comprehensive understanding of how different drying processes affect the quality of dried fruit products. Additionally, larger‐scale sensory evaluations with trained panels would yield more reliable consumer preference data. Investigating the nutritional impacts of different drying methods, particularly concerning bioactive compounds, would also contribute to a deeper understanding of how these processes influence the overall quality and health benefits of dried fruit products. In general, this study advances the effort to improve the quality and marketability of dried fruit products. By optimizing ripening stages and drying techniques, researchers and industry professionals can create innovative, consumer‐oriented products that also align with sustainability goals. Supporting Information.

## AUTHOR CONTRIBUTIONS

**Messenbet Geremew Kassa**: Investigation; formal analysis; writing—original draft. **Desye Alemu Teferi**: Resources; writing—review and editing; software.

## CONFLICT OF INTEREST STATEMENT

The authors declare no conflicts of interest.

## ETHICS STATEMENT

The research in this study strictly adhered to the ethical guidelines established by the Institutional Review Board (IRB) of Injibara University. Prior to commencing the research, formal approval was obtained from the university's IRB, under the reference number IN/U/C/A/F/C/S/327/09. All procedures involving human participants were meticulously conducted in accordance with the ethical principles outlined by the IRB. The study protocol underwent a comprehensive review and received approval from the university's Ethics Committee IRB. Informed consent was diligently obtained from all participants before their involvement in the study, ensuring their voluntary participation and a clear understanding of the research objectives. All authors verify that the study in the manuscript does not involve any human or animal trial experiments. However, in the case of the sensory, all participants who underwent sensory evaluation signed a written informed consent to the protocol approved by the Ethiopian Health Research Ethics Board, National Ethics Committee (NHREB), following the ethical standards as laid down in the Declaration of Helsinki and its later amendments or comparable standards.

## Data Availability

All the data used in this research are included in the article.
